# Endogenous VEGF Is Required for Visual Function: Evidence for a Survival Role on Müller Cells and Photoreceptors

**DOI:** 10.1371/journal.pone.0003554

**Published:** 2008-11-03

**Authors:** Magali Saint-Geniez, Arindel S. R. Maharaj, Tony E. Walshe, Budd A. Tucker, Eiichi Sekiyama, Tomoki Kurihara, Diane C. Darland, Michael J. Young, Patricia A. D'Amore

**Affiliations:** 1 Schepens Eye Research Institute, Harvard Medical School, Boston, Massachusetts, United States of America; 2 Department of Ophthalmology, Harvard Medical School, Boston, Massachusetts, United States of America; 3 Department of Pathology, Harvard Medical School, Boston, Massachusetts, United States of America; 4 University of North Dakota, Grand Forks, North Dakota, United States of America; University of Sydney, Australia

## Abstract

**Background:**

Vascular endothelial growth factor (VEGF) is well known for its role in normal and pathologic neovascularization. However, a growing body of evidence indicates that VEGF also acts on non-vascular cells, both developmentally as well as in the adult. In light of the widespread use of systemic and intraocular anti-VEGF therapies for the treatment of angiogenesis associated with tumor growth and wet macular degeneration, systematic investigation of the role of VEGF in the adult retina is critical.

**Methods and Findings:**

Using immunohistochemistry and Lac-Z reporter mouse lines, we report that VEGF is produced by various cells in the adult mouse retina and that VEGFR2, the primary signaling receptor, is also widely expressed, with strong expression by Müller cells and photoreceptors. Systemic neutralization of VEGF was accomplished in mice by adenoviral expression of sFlt1. After 14 days of VEGF neutralization, there was no effect on the inner and outer retina vasculature, but a significant increase in apoptosis of cells in the inner and outer nuclear layers. By four weeks, the increase in neural cell death was associated with reduced thickness of the inner and outer nuclear layers and a decline in retinal function as measured by electroretinograms. siRNA-based suppression of VEGF expression in a Müller cell line in vitro supports the existence of an autocrine role for VEGF in Müller cell survival. Similarly, the addition of exogenous VEGF to freshly isolated photoreceptor cells and outer-nuclear-layer explants demonstrated VEGF to be highly neuroprotective.

**Conclusions:**

These results indicate an important role for endogenous VEGF in the maintenance and function of adult retina neuronal cells and indicate that anti-VEGF therapies should be administered with caution.

## Introduction

The retina is one of the body's most metabolically demanding tissues. To ensure adequate nutrient and oxygen delivery, the retina is supplied by two independent vascular beds, the inner retinal vasculature and outer choroidal circulation. These vascular beds have distinct morphologic properties; the inner retinal vasculature is the site of a blood-tissue barrier and is characterized by highly impermeable vessels, whereas the choriocapillaris consists of a highly fenestrated capillary plexus. Vascularization of the retina (reviewed in [1]) occurs late in gestation and is restricted to the inner retina. During development, vascular endothelial growth factor (VEGF) is expressed by astrocytes in the retinal ganglion cell layer (GCL), by cells of inner nuclear layer (INL), Müller cells, and retinal pigment epithelial cells (RPE) [2], [3]. Targeted deletion of VEGF in the RPE results in failure of choroidal development and loss of visual function [4]. Whereas formation of the choriocapillaris appears to be independent of hypoxia [4], formation of the inner retinal vasculature requires induction of the hypoxia inducible factor-1β [5]. Hyperoxia during retinal development suppresses VEGF production resulting in reduced vascular development [5], as is seen in retinopathy of prematurity [6]. Furthermore, normal retinal vascularization requires the coordinated expression of specific VEGF isoforms, which differ in their solubility and binding to VEGF receptors [7].

In addition to its role in developmental retinal vascularization, VEGF is upregulated in several ocular pathologies, including wet age related macular degeneration (AMD) [8] and proliferative diabetic retinopathy [9]. In AMD, injury to the RPE is thought to result in increased VEGF expression. Coupled with damage to Bruch's membrane, this leads to proliferation of choroidal vessels, which invade the subretinal space [10]. In proliferative diabetic retinopathy, the neovascularization that arises from the inner retina is mediated by hypoxia-induced VEGF (reviewed in [11]). The involvement of VEGF in AMD has led to the Food and Drug Administration approval of two intraocular anti-VEGF drugs, pegaptanib sodium (Macugen, OSI/Eyetech Pharmaceuticals, NY) [12], an aptamer which was reported to inhibits only VEGF165, and ranibizumab (Lucentis, Genentech) [13], a Fab fragment of the humanized monoclonal VEGF antibody bevacizumab (Avastin, Genentech) which neutralizes all VEGF isoforms.

While the role of VEGF in developmental and pathologic retinal vascularization is well understood, its function in the normal adult is unclear. We and others have previously reported that VEGF is expressed in the adult retina by RPE [3] as well as by neuronal and glial cells [14], [15], [16], [17]. Although initially thought to be endothelial-specific, VEGF has been shown to target a variety of non-vascular cells (reviewed in [18]) including, neural stem cells [19], ependymal cells [20] and neuronal cells of the central nervous system [21], [22]. In addition, VEGF neutralization in humans via intraocular injection of Macugen and Avastin is associated with an increased incidence of retinal tears [23], [24]. Similarly, preeclampsia, which is mediated in part by elevated circulating levels of a soluble form of VEGF receptor 1 called sFlt1 leading to systemic VEGF neutralization [25], is also associated with ocular symptoms including RPE lesions, choroidal ischemia and retinal detachments [26], [27].

Given the expression of VEGF and VEGF receptor 2 (VEGFR2) in the adult retina, and the constitutive activation of the receptor, we hypothesized that VEGF plays a role in maintenance and function of the adult retina. We report that, in addition to the choriocapillaris and retinal vasculature, VEGFR2 is expressed by adult photoreceptor cells and Müller cells. While systemic neutralization of VEGF in the mouse did not lead to detectable changes in retinal vascular perfusion or permeability, there was significant cell death in the INL and outer nuclear layer (ONL) as well as loss of visual function. A direct survival function for VEGF was demonstrated using a Müller cell line as well as primary photoreceptor cells and sheets. Taken together, these observations implicate VEGF in maintenance of the adult neural retina.

## Results

### VEGF and VEGFR2 expression in adult retina

**Figure 1:**
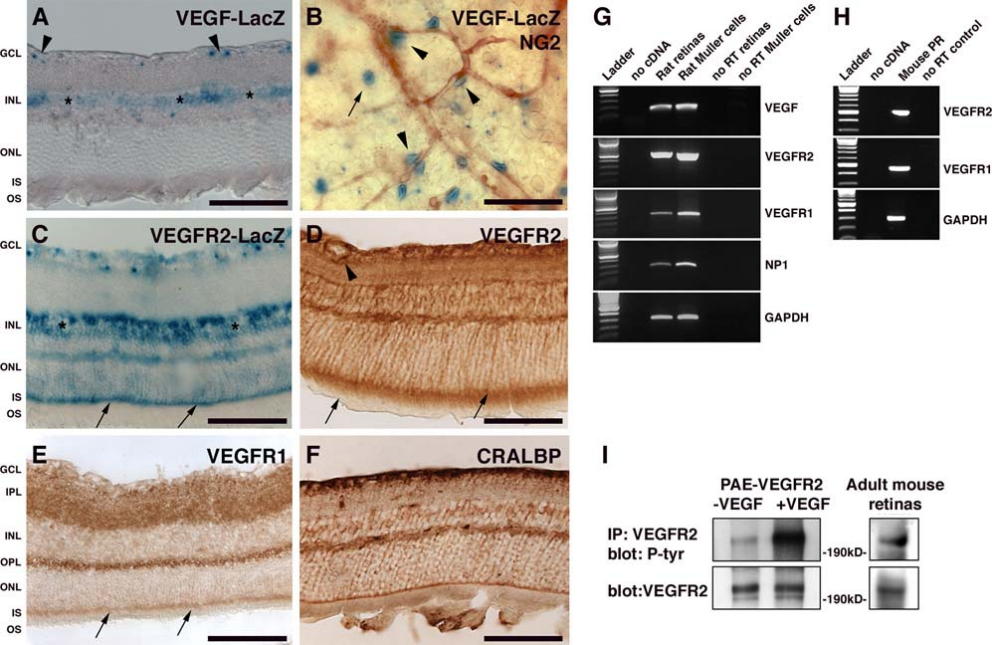
VEGF signaling in the adult retina. (A) Sections of eyes from adult *VEGF-lacZ* mice stained for LacZ using x-gal (blue) revealed VEGF expression in the GCL (arrowheads) and INL layer (asterisks). (B) Flat-mounted retinas from adult VEGF-lacZ mice stained for lacZ (blue) and NG2 (brown) demonstrated VEGF expression by NG2-positive cells associated with microvessels (arrowhead) and by some astrocytes (arrow) in the GCL. (C) Sections of eyes from adult *VEGFR2-lacZ* mice stained for lacZ revealed expression of VEGFR2 in the GCL, in the INL (asterisks), and in the photoreceptors where a strong lacZ staining was observed in the inner segments (arrows). (D) Immunohistochemistry for VEGFR2 in sections of adult retina revealed expression in vascular cells (arrowhead), in Müller cells processes, and in the IS of the photoreceptors (arrows). (E) Immunohistochemistry for VEGFR1 revealed a spotty expression in the IPL and OPL. VEGFR1 was also detected in the photoreceptor IS. (F) Staining of Müller cells using CRALBP revealed an expression pattern similar to VEGFR2 (compare with D). (G) Expression of VEGF and its receptors, VEGFR1 and VEGFR2, by Müller cells was confirmed by RT-PCR of RNA from adult rat retina and isolated rat Müller cells. (H) Expression of VEGFR1 and VEGFR2 in photoreceptors (PR) isolated from adult mouse retinas. (I) Immunoprecipitation (IP) of VEGFR2 from pooled adult mouse retinas, followed by immunoblotting for phosphorylated tyrosine revealed VEGFR2 expression (bottom panel) and activation (top panel) in the adult retina. As a control, lysates of porcine aortic endothelial (PAE) cells overexpressing VEGFR2 either untreated or stimulated with VEGF were immunoprecipitated and immunoblotted as described above. GCL: ganglion cell layer, IPL: inner plexiform layer, INL: inner nuclear layer, OPL: outer plexiform layer, ONL: outer nuclear layer, IS: inner segment, OS: outer segment. Scale bar is 100 µm.

We have previously shown that VEGF is expressed by embryonic and adult RPE [3]. To examine VEGF expression patterns in adult eye, we used mice heterozygous for the LacZ reporter gene with a nuclear localization signal under the control of the VEGF promoter (28). Here we show that VEGF is also expressed by cells in the GCL and INL in the adult mouse retina (Figure 1A, arrowheads and asterisks, respectively). To determine the identity of the cells expressing VEGF in the GCL, flat-mounted retinas from adult VEGF-LacZ mice [28] were co-labeled for lac-Z and for NG2, the chondroitin sulfate proteoglycan, as a pericyte marker [29]. There were two distinct populations of Lac-Z positive cells. One was pericytes, identified by their expression of NG2 and their characteristic abluminal association with the microvessels (Figure 1B arrowheads). The second population of VEGF-expressing cells in the innermost retinal layer were astrocytes (Figure 1B arrow and Figure S1). In the retina, astrocytes are characterized by cell bodies that are located in the intercapillary spaces but with processes closely associated with the microvessels. Immunohistochemical localization of the astrocyte marker, glial fibrillary acidic protein (GFAP) [30], on VEGF-LacZ adult retina confirmed this second VEGF expressing cell population to be astrocytes (Figure S1). This pattern of VEGF expression in the GCL is consistent with our previous observations in postnatal retinal development [2].

To precisely identify the cellular targets of VEGF, we examined sections of retinas from heterozygous VEGFR2-lacZ mice where the 5′ region of the *VEGFR2* gene was replaced with a promoterless *lacZ* gene from *Escherichia Coli* [31]. β-gal staining revealed that VEGFR2 was expressed by vascular cells in the GCL (Figure 1D, arrowhead) and INL as well as by the photoreceptors (ONL and inner segments) (Figure 1C, arrows). Consistent with the lac-Z staining, VEGFR2 expression, determined by immunochemistry, was observed throughout most of the neural retina (Figure 1D). Strong VEGFR2 immunoreactivity was noted in retinal microvasculature (Figure 1D, arrowhead). The comparison of VEGFR2 expression pattern with the Müller cell marker, cellular retinaldehyde binding-protein (CRALBP) [32] (Figure 1F), indicated expression of VEGFR2 by Müller cells. Müller cell nuclei are localized in the INL and they project processes that extend from the inner limiting membrane to the outer part of the ONL. Immunodetection of VEGFR1 revealed a punctuate staining of the inner and outer plexiform layers as well as of the inner segments (IS) of photoreceptors (Figure 1E). RT-PCR of adult rat retina and purified Müller cells revealed enrichment of RNA for VEGF as well as VEGFR1, VEGFR2 and neuropilin-1 (NP1) signals in Müller cells, suggesting that Müller cells are a significant source of VEGF and its receptors in the retina. The localization of VEGF receptors in the IS of the photoreceptors was confirmed by RT-PCR analysis of VEGFR1 and VEGFR2 on freshly isolated adult mouse photoreceptors (Figure 1H).

Examination of the activation status of VEGFR2, the main signaling receptor for VEGF, was accomplished by immunopreciptation from pooled adult retina lysates, followed by immunoblotting for phosphorylated tyrosine. Results of this analysis indicated that VEGFR2 is constitutively activated in the adult retina (Figure 1I), an observation that is consistent with a role of VEGF in adult retina homeostasis.

### Systemic VEGF neutralization does not affect retinal vasculature

**Figure 2 pone-0003554-g002:**
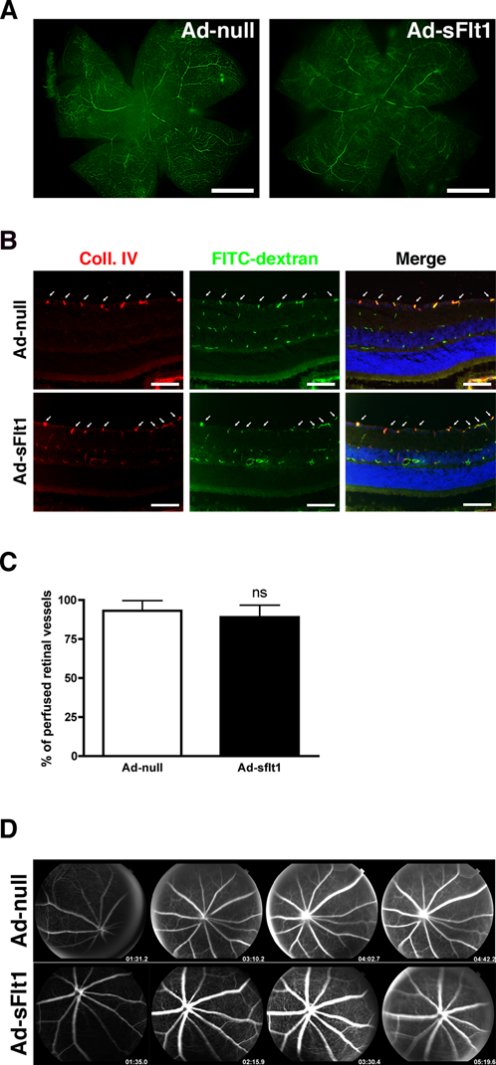
VEGF neutralization does not affect retinal vascular perfusion or permeability. Mice infected with Ad-null or Ad-sFlt1 for 14 days were perfused with h.m.w fluorescein dextran (green), then eyes were dissected and (A) flat mounted or (B) sectioned and immunostained for collagen IV, and visualized with a Cy3-conjugated secondary antibody (red). (A) Flat mounts of retinas from mice expressing sFlt1 perfused with fluorescein dextran shows no gross abnormalities or perfusion defects, and no changes in capillary density in the inner retina compared to the control mice expressing Ad-null (n = 6 mice/condition). (B) Immunofluorescent localization of collagen IV in sections of fluorescein-perfused retinas revealed nearly complete overlay of FITC-dextran and collagen IV of the inner retinal vessels in both Ad-null and Ad-sFlt1 (arrows, third column). (C) Quantification of fluorescein- and collagen IV-positive vessels of the innermost retinal vasculature in Ad-sFlt1 (n = 5) compared to Ad-null (n = 4) showed no reduction in the number of perfused vessels, indicating the absence of vascular damage. (D) Fluorescein angiography of experimental mice seven days after infection showing no opacity in the vitreous 4-5 min after injection in either group, indicating no change in retinal vascular permeability (n = 6 mice/condition). Scale bar is 1 mm in A and 100 µm in B.

To elucidate the function of VEGF in the adult retina, we neutralized VEGF systemically using an adenovirus expressing sFlt1 (Ad-sFlt1). sFlt1, a soluble form of VEGFR1 produced by alternative mRNA splicing, is a potent VEGF inhibitor [33]. As prior reports indicate a role for VEGF in maintenance of the microvasculature of various organs, including the trachea, pancreas and thyroid [34], we first sought to determine if systemic sFlt1-expression led to any changes in retinal vessels. Examination of retinal flat mounts from fluorescein-perfused mice expressing-sFlt1 for 14 days revealed no gross changes in the retinal vasculature (Figure 2A). Collagen IV immunostaining of retinal sections from fluorescein-dextran perfused mice revealed normal perfusion in the retinas of mice that had expressed sFlt1 for 14 days compared to controls (Figure 2B and C). NG2 staining of retinal flat mounts from sFlt1-expressing mice showed no apparent changes in pericyte association to the retinal microvessels (Figure S2).

**Figure 3 pone-0003554-g003:**
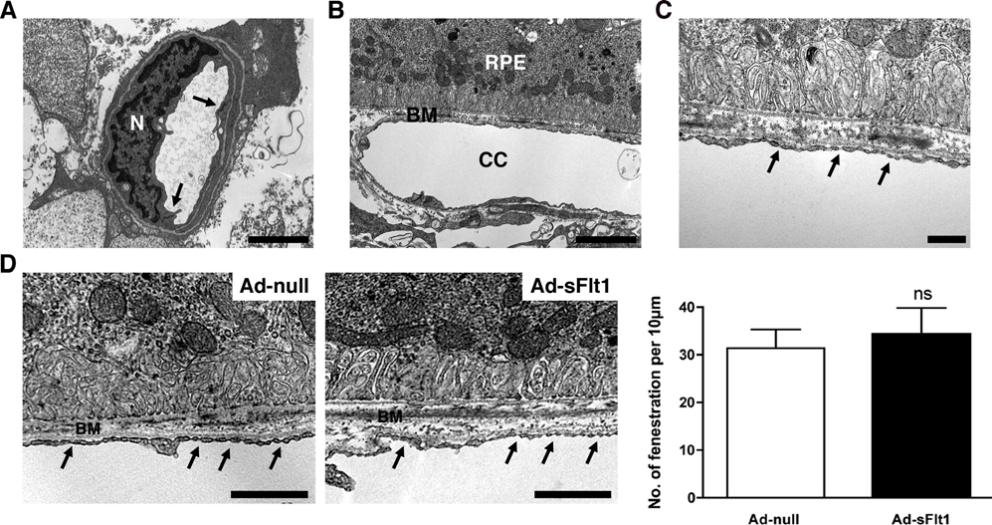
Systemic VEGF blockade does not alter retinal vessels ultrastructure or choriocapillaris fenestration. (A) TEM of a microvessel located in the GCL of sFlt1 expressing mouse at day 14 showed a normal ultrastructure of the endothelial nuclei and cytoplasm. Characteristically electron -dense tight junctions were well defined (arrows). (B) TEM micrograph of the outer segment of a retina, 14 days post-infection of Ad-sFlt1, revealed a normal RPE-choroid complex. The RPE basal infoldings were dense and well associated with the Bruch's membrane (BM). The choriocapillaris showed no thickening of the membrane. No sign of thrombosis were detected. (C) Higher magnification of the choriocapillaris revealed numerous fenestrations on the endothelial membrane facing the RPE (arrows). (D) Endothelial fenestrations of the choriocapillaris (arrows) were quantified at 28 days post-infection on TEM micrographs (n = 3 mice/condition). No change in the number of choriocapillaris fenestration was observed between Ad-null and Ad-sFlt1 mice. Scale bar is 5 µm in A–B, 0.5 µm in C and 1 µm in D.

As it has been previously shown that systemic neutralization of VEGF leads to increased permeability in the lung and kidney of sFlt1-expressing rats [35] and in the brain of sFlt1-expressing mice [20], we sought to determine if systemic VEGF neutralization would affect the permeability of the retinal vasculature. The vessels of the retinal vasculature have properties similar to the blood brain barrier, and as such, are relatively impermeable [36]. Fluorescein angiography of the retina demonstrated no differences in fluorescein leakage between mice expressing Ad-null or Ad-sFlt1 for seven days (Figure 2D). Transmission electron microscopic examination of the retinal vasculature did not reveal structural alterations in the inner retina capillaries or the choriocapillaris (Figure 3A and B). The capillaries of the GCL of sFlt1-expressing mice displayed normal morphology with well-defined extracellular space and basement membrane, few cytoplasmic vacuoles and normal tight junctions (Figure 3A).

Similarly, there were no structural abnormalities of the RPE-choriocapillaris complex. The Bruch's membrane of sFlt1-expressing mice appeared normal with well defined layers and no apparent thickening. No sign of thrombosis could be detected (Figure 3B). The choriocapillaris retained their characteristic fenestrated phenotype (Figure 3C). Electron microscopic quantification of fenestrations in the choriocapillaris endothelium of mice expressing sFlt1 for 28 days showed no difference compared to the Ad-null infected controls (Figure 3D).

### VEGF neutralization leads to neuroretinal cell apoptosis and loss of retinal function

**Figure 4 pone-0003554-g004:**
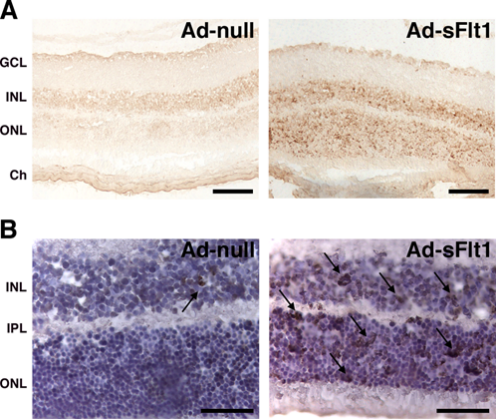
VEGF neutralization leads to increased retina cell death. (A) TUNEL staining showed markedly increased apoptosis in both the INL and ONL of Ad-sFlt1 mice 14 days post-infection (n = 4 mice/condition). (B) Higher magnification images of retinal sections of sFlt1-expressing mice stained for TUNEL and counterstained with hematoxylin revealed an significant increase in the number of apoptotic cells in the INL and ONL. GCL: ganglion cell layer, INL: inner nuclear layer, ONL: outer nuclear layer, OPL: outer plexiform layer, Ch: choroid. Scale bars are 100 µm in A and 50 µm in B.

**Figure 5 pone-0003554-g005:**
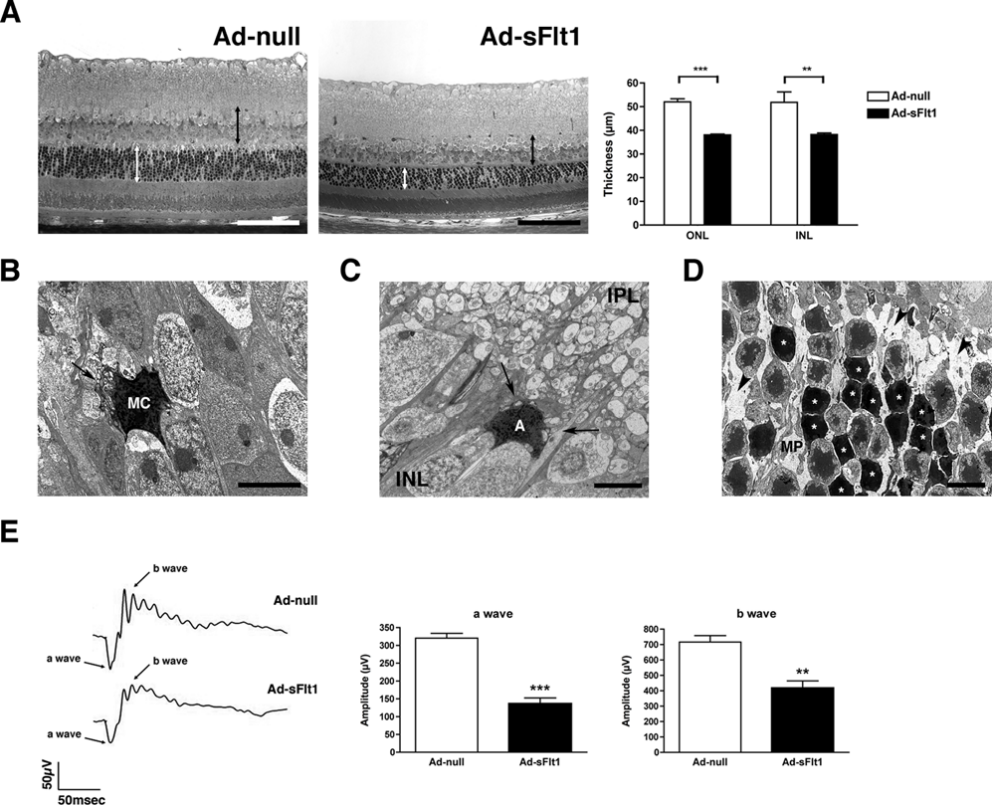
Decreased retina thickness and loss of visual function in sFlt1 expressing mice. (A) Semi-thin epon sections from retinas of experimental mice expressing sFlt1 for 28 days showed substantial thinning of the INL (black double arrowheads line) and the ONL (white double arrowheads line) (n = 3 for Ad-null and n = 4 for Ad-sFlt1). (B–D) TEM of retinas from sFlt1 expressing mice at 28 days. (B–C) Micrographs taken in the INL region revealed apoptotic Müller cells (MC) and amacrine cells (A). Both cells displayed condensation of the chromatin, membrane swelling and rupture mitochondria (arrows). (D) In the ONL, a high percentage of photoreceptors appeared apoptotic (white asterisk). Cell death left numerous empty spaces containing membranous debris (arrowhead). Processes from the Müller cells that normally fill the intercellular space appeared shrunken (MP). (E) Scotopic ERG recordings of experimental mice 28 days post-infection using a flash intensity of +10 dB revealed a marked reduction of both a- and b-wave amplitudes in sFlt1-expressing mice (n = 6 for Ad-null and n = 10 for Ad-sFlt1). GCL: ganglion cell layer, INL: inner nuclear layer, ONL: outer nuclear layer, OPL: outer plexiform layer, Ch: choroid. Scale bars are 100 µm in A and 5 µm in B to D.

Examination of retinal cryosections from mice expressing sFlt1 for 14 days revealed significant apoptosis in both the INL and ONL (Figure 4A–B). Retinal damage is generally associated with Müller cell activation that is marked by upregulation of GFAP [37]. However, there was no increase in GFAP expression in Müller cells of mice expressing sFlt1 for 14 days (Figure S3), suggesting that the observed cell death is not the consequence of any ongoing retinal injury. The increased cell death resulted in a significant reduction in the thickness of the INL and ONL by day 28 (Figure 5A).

The INL is comprised primarily of Müller and neural cells, including bipolar, horizontal and amacrine cells, whereas the ONL contains photoreceptor cell bodies. Electron micrographs revealed typical signs of apoptosis in various cells of the INL. Apoptotic Müller (Figure 5B) and amacrine cells (Figure 5C) could be identified based on their location and morphological characteristics [38]. Both cells showed chromatin condensation, membrane swelling and ruptured mitochondria (Figure 5B and C). Cell death was even more evident in the ONL where a high number of photoreceptor cell bodies displayed cellular shrinkage, and chromatin condensation (Figure 5D). The combination of photoreceptor cell death and retraction of Müller cells processes (MP), which are normally closely associated with the photoreceptors, left large empty spaces containing cellular debris (Figure 5D).

Visual dysfunction resulting from the increased cell death observed in sFlt1 expressing mice was assessed using electroretinogram (ERG) recordings. The ERG results from the electric current created by light-induced activity of neuronal and glial cells [39]. ERG recording revealed a significant reduction of both a- and b-wave amplitude (Figure 5E). Reduction of more than half of the a-wave amplitude is indicative of a considerable loss of photoreceptor function and is consistent with our previous observations. The reduction of the b-wave is a sign of an altered photoreceptor post-synaptic cascade that reflects the increase in photoreceptor apoptosis. A possible contribution of bipolar cell dysfunction to the reduced b-wave could not be excluded.

### Autocrine VEGF signaling plays a role in Müller cell survival

**Figure 6 pone-0003554-g006:**
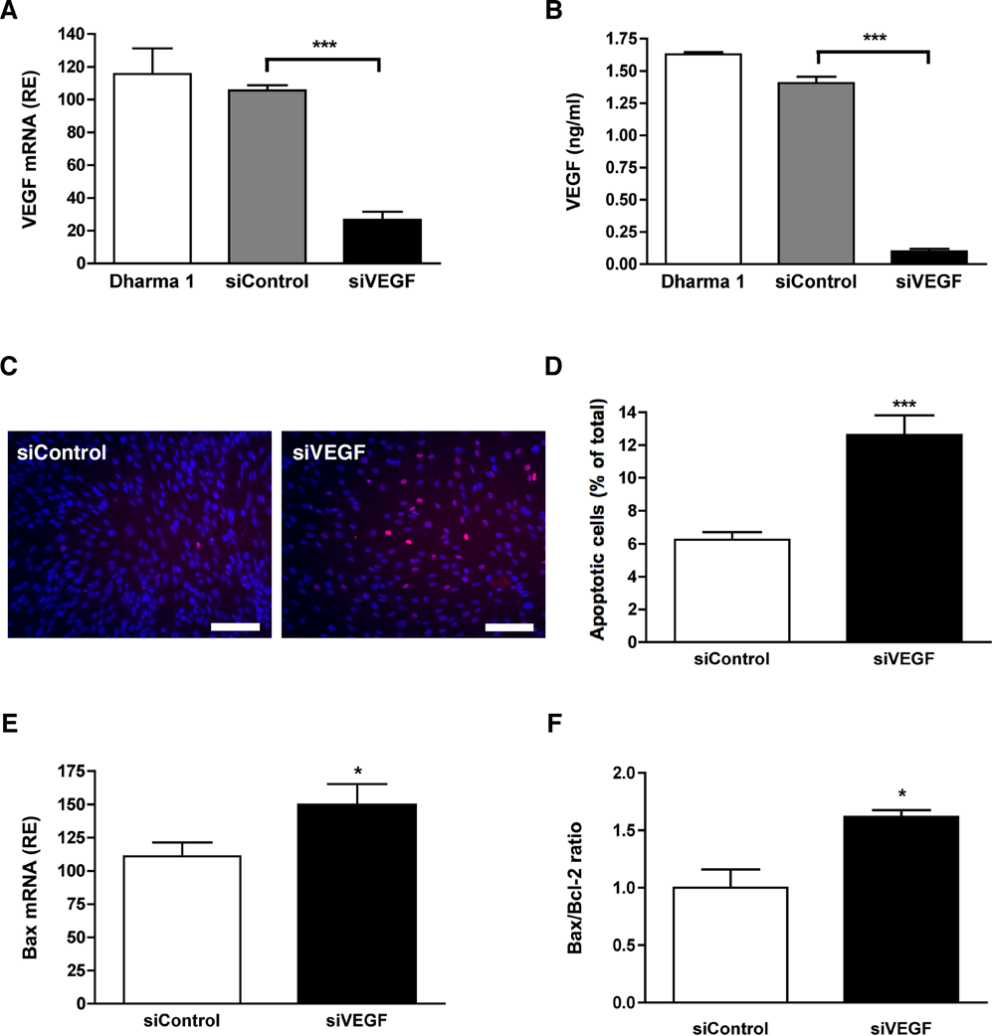
Inhibition of the autocrine VEGF signaling in Müller cells leads to increased cell death. VEGF expression by MIO-M1 Müller cells was inhibited by transfection of siRNA into sub-confluent cells. (A–B) Seventy-two hr following transfection of the control and VEGF siRNA, VEGF mRNA levels were determined by qPCR (A) and VEGF protein secretion was quantified by ELISA (B). siVEGF transfection led to a 75% inhibition of VEGF mRNA and more than 90% reduction of VEGF protein (n = 3). (C) Inhibition of VEGF was associated with an increase in TUNEL-positive cells after three days of culture in serum-free conditions (n = 4). (D) Quantification of cell death by FACS analysis demonstrated a doubling of the number of annexin-V positive apoptotic cells in siVEGF transfected cells compared to siControl (n = 3). (E) Increased apoptosis was accompanied by a significant up-regulation of the pro-apoptotic gene, *bax*, and by the increase of the *Bax*/*Bcl-2* ratio (F). Scale bar is 100 µm.

In light of the co-expression of VEGF and its receptors by Müller cells, we investigated whether the disruption of a VEGF autocrine pathway on Müller cells was mediating the increased cell death in the INL of sFlt1 expressing mice. Because of the technical difficulty to isolate sufficient numbers of differentiated Müller cells, we used the Müller cell line, MIO-M1. MIO-M1 is a spontaneously immortalized human Müller cell line that retains most of the characteristic Müller cell morphology, protein expression and function [40]. We used siRNA mRNA to alter autocrine VEGF signaling by inhibiting its autonomous expression. Transfection of siVEGF led to a 76% decrease in VEGF mRNA (Figure 6A) and a 94% reduction of secreted VEGF protein (from 1.63 ng/ml±0.01 to 0.1 ng/ml±0.01) at day 3 (Figure 6B). Inhibition of VEGF expression under serum-free conditions led to an increase in the number of TUNEL-positive MIO-M1 cells (Figure 6C). Quantification of apoptosis by FACS analysis revealed a doubling in the number of apoptotic cells in siVEGF-transfected cells compared to cells transfected with control siRNA (Figure 6D).

Apoptosis is tightly controlled by the balance between pro- and anti-apoptotic genes of the Bcl-2 family. Changes in the relative expression of the Bcl-2 family members in favor of pro-angiogenic genes will ultimately decide cell fate [41]. Therefore, we determined the expression level of the pro-apoptotic gene *Bax* and anti-apoptotic genes *Bcl-2* and *Bcl-x_L_* by quantitative PCR. Inhibition of VEGF expression by MIO-M1 cells resulted in the up-regulation of the pro-apoptotic gene *Bax* (Figure 6E), leading to a significant increase in the *Bax/Bcl-2* expression ratio (Figure 6F). No significant changes in *Bcl-2* and *Bcl-x_L_* expression were detected (data not shown).

### VEGF is a direct survival factor for photoreceptors

**Figure 7:**
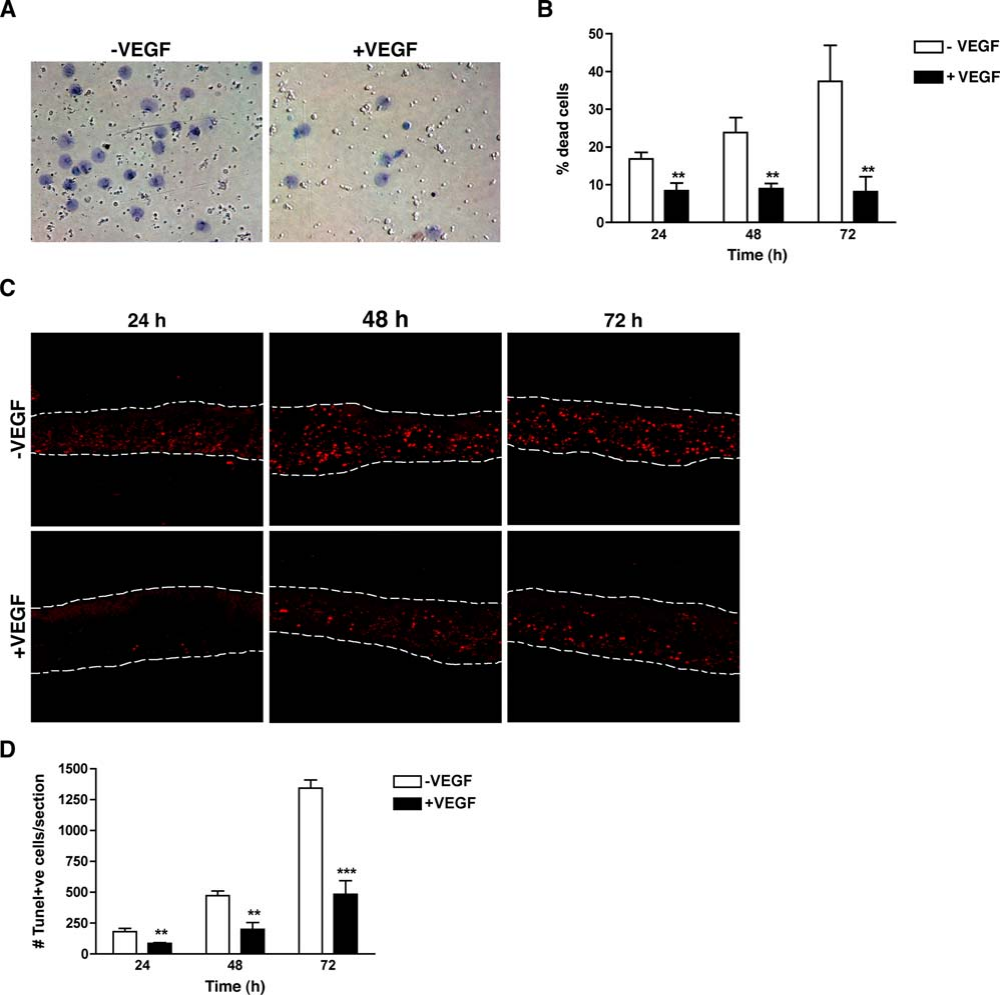
VEGF is a direct survival factor for photoreceptors. (A) Photoreceptors isolated from adult C57BL/6J mice were cultured on laminin-coated wells in absence or presence of 10 ng/ml VEGF165 for up to 72 hr. Addition of 10 ng/ml of VEGF165 decreased the number of dead cells identified by trypan blue staining. (B) Quantification of the percentage of dead photoreceptors based on the trypan blue exclusion assay revealed a significant protection from apoptosis by VEGF165 (n = 9). (C) ONL explants containing only the photoreceptor nuclei and IS were cultured in basal medium±10 ng/ml of VEGF165 for 24, 48 and 72 hr. Apoptotic cells were detected by TUNEL staining. (D) Quantification of TUNEL-positive cells per sheet revealed a significant decreased in the number of apoptotic photoreceptors in presence of VEGF (n = 6).

Based on our observation that photoreceptors express VEGF receptors, we used freshly isolated post-mitotic mouse photoreceptors to determine if VEGF could directly influence photoreceptor survival. Photoreceptor sheets were isolated by mechanical fractionation of flat-mounted retinas (Figure S4). Purity of the preparation was controlled by co-immunodetection of the photoreceptor marker, recoverin, and the Müller cell markers, GFAP and glutamine synthetase (GS) (Figure S5). Isolated photoreceptor cells cultured in absence of serum died rapidly (as measured by Trypan blue exclusion) (Figure 7A). However, the addition of 10 ng/ml of VEGF165 was sufficient to rescue the photoreceptors, so that at 72 hr there were only 8.2% apoptotic cells in the presence of VEGF compared to 37.4% in the absence of VEGF (Figure 7B). This pro-survival function of VEGF was confirmed using photoreceptor explants in which 60 µm sections containing only the photoreceptor cell bodies and their inner segments were cultured in presence or absence of VEGF165. As observed with isolated photoreceptor cells, the absence of serum resulted in a time-dependent increase in apoptosis while the addition of VEGF led to significant protection against apoptosis (Figure 7C–D).

## Discussion

There is growing evidence that VEGF effects are not specific to the vasculature. A variety of non-vascular cells express VEGFR2, the primary VEGF signaling receptor, and VEGF has been shown to act on these cells to mediate proliferation, differentiation and/or survival (for review see [18]). Previous studies reported the expression of VEGFR2 by glial and neuronal cells of the retina [14], [42], [43], [44] but VEGFR2 expression by photoreceptors has not been definitively established [43], [45]. The combined use of VEGFR2-LacZ mice and immunohistochemistry for VEGFR2 allow us to unequivocally demonstrate the expression of VEGFR2 on Müller cells and photoreceptors. The wide expression of VEGF and VEGFR2 in adult retina and the finding that VEGFR2 is constitutively phosphorylated strongly indicates a role for VEGF signaling in retina homeostasis. We therefore elected to assess the role of VEGF in the normal retina by systemic neutralization. Systemic blockade was selected over intravitreal injection because these studies are intended to assess the effect of sustained and long-term VEGF inhibition in adult mice.

The observation that Müller cells, which express both VEGF as well as its receptors, VEGFR1 and VEGFR2, undergo apoptosis in mice with systemic VEGF neutralization suggested an autocrine role for VEGF in Müller cells *in vivo*. This concept is strongly supported by the finding that siRNA suppression of VEGF expression in cultured Müller cells leads to a significant increase in cell death. Co-expression of VEGF and VEGFR2 has been observed in many tumor cells [46], [47] but only in a few normal cell types, such as podocytes [48], skeletal muscle [49], aortic endothelium [50], [51], and RPE [3]. However, a role for VEGF autocrine pathways in tissue homeostasis has only been demonstrated *in vivo* in the endothelium, where cell-autonomous signaling of VEGF appears to be required for endothelial cell survival [51].

Our data showed that VEGF autocrine signaling promotes Müller cell survival in serum-free conditions through the control of pro-apoptotic *Bax* gene expression. Interestingly, no changes in the level of expression of anti-apoptotic genes *Bcl-2* and *Bcl-x_L_* were observed after alteration of VEGF autocrine signaling. *Bax* constitutes one of the main transcriptional targets of p53 in neuronal and glial cells [52] suggesting that VEGF autocrine signaling regulates Müller cell apoptosis through the control of p53 expression/activation and *Bax* expression.

The physical association between the photoreceptors that express VEGFR2 and the Müller cells that express VEGF points to a paracrine interaction in which Müller cells provide VEGF as a neurotrophic signal to photoreceptors. The dramatic increase in apoptosis associated with visual dysfunction observed in mice expressing sFlt1 is consistent with this suggestion. Using purified photoreceptors, we were able to demonstrate a direct function of VEGF on photoreceptor survival independent of an effect on accessory cells. Müller cells act to support retinal neurons by providing structural support for the retina and regulating ion and neurotransmitter levels in the extracellular space [53]. In addition, they produce a variety of neurotrophic factors, including brain-derived neurotrophic factor (BDNF) [54], nerve growth factor (NGF) [55] and neurotrophins (NT-3 and NT-4/5) [56]. Our findings add VEGF to this list. Though retinal pigment epithelial cells (RPE) also produce VEGF in the adult [3], they are unlikely to be a primary source of VEGF for the photoreceptors because of their distance from the photoreceptor cell bodies. Moreover, RPE have been shown to secrete VEGF basally, presumably in support of the underlying fenestrated choriocapillaris [4]. In addition to supporting photoreceptors, Müller cells have been to shown to maintain adult retinal ganglion cell (RGC) survival in vitro [57] and the fact that RGC express VEGFR2 [43] suggests that Müller cell-derived VEGF also plays a supporting role for RGCs. In support of this, it has been demonstrated that long-term VEGF (8 wk) blockade in the rat leads to RGC apoptosis [43].

The requirement of glial and neuronal cells for VEGF as a survival factor under normal conditions is one of the most intriguing findings of our study. Müller cells and neurons produce numerous neurotrophic factors to support the retina [58], [59]. Dependence on VEGF signaling may be due to the high metabolic rates of retinal neurons and their extreme sensitivity to hypoxia [60]. As VEGF expression is tightly controlled by oxygen tension and glycemia level [61], [62], VEGF would be the ideal candidate to support retina homeostasis during the early phase of an oxidative or ischemic insult. Indeed, VEGF has been shown to protect neuronal cells of the central nervous system (CNS) against apoptosis induced by serum deprivation or hypoxia [63]. The VEGF-dependent paracrine relationship between the Müller cell and photoreceptors that we have revealed here parallels the trophic association between Schwann cells and neurons in the CNS [64].

We have previously observed VEGF expression in virtually all vascularized adult tissues [50], [65] and we and others have hypothesized that VEGF plays an important role in the maintenance of the adult vasculature [34], [50]. In support of this, relatively short-term systemic VEGF neutralization has been shown to lead to the selective regression of some fenestrated microvessels [34], leading to the hypothesis that these fenestrated vessels are particularly dependent on VEGF for their survival. In fact, we have shown that nearly all fenestrated vessels, including for example the glomerulus of the kidney and the chroroid plexus in the brain, lie in close proximity to epithelial cells that express high levels of VEGF [50]. Studies *in vitro* and *in vivo* [66], [67] have demonstrated that ectopic application of VEGF can induce the formation of fenestrations. Interestingly, our analysis did not reveal an effect of systemic VEGF neutralization on the fenestrations of the choriocapillaris. A recent report in which intravitreal administration of Avastin in a primate model led to early loss of fenestration with rapid recovery in spite of the prolonged Avastin half-life [68], leads us to speculate that the loss of fenestrations is a transient effect that one might not expect to be detectable at this later (28 days post infection) time point.

Our observation of relatively low levels of VEGF in the innermost retinal layers and the fact that systemic VEGF expression does not influence the inner retinal vasculature, which is not fenestrated but is the site of the blood neural barrier, are consistent with the idea that high local VEGF levels reflect a particular dependence on VEGF, which renders them particularly sensitive to systemic VEGF blockade. Consistent with this notion, the microvasculature of the brain, which has relatively low levels of VEGF in the adult [50], was not influenced by 14 days of systemic VEGF inhibition [34]. In addition to the relatively low levels of local VEGF, the microvasculatures of the retina and brain are characterized by high ratio of pericyte to endothelial cells [69]. In light of the evidence that pericyte association with EC mediates vessel stabilization, it is reasonable to suggest that the microvasculature of the CNS is particularly stable. On the other hand, it is difficult to evaluate the extent of VEGF neutralization at the level of the retinal microvasculature by systemically expression of sFlt1. Based on our previous extensive use of endothelial cell-pericytes cocultures [70], [71] and the documented close association between endothelial cells and pericytes in the retina [72], [73], we suspect that the VEGF produced by pericytes to support retinal capillary endothelial cells (Figure 1D in this paper and [2]) may be relatively less accessible to soluble Flt-1 and thus may account for the lack of an effect of VEGF neutralization on the inner retinal vessels in the time frame of this study.

Our results have obvious implications for therapeutic use of anti-VEGF, particularly as it relates to the eye, as we observed that VEGF neutralization interferes with endogenous survival signals, leading to unexpected neural toxicity. On the other hand, however, since the adult retina expresses VEGF in the absence of active neovascularization, it is clear that the locally produced VEGF mediates neural protection without leading to vessel permeability or growth. The absence of active vessel proliferation/permeability in the normal retina in the presence of endogenous VEGF may be due to the dose, localization and/or the presence of opposing factors, such as TGF-β [74], [75] or angiopoietin-1 [76]. However, it does indicate that, with appropriate delivery and/or dosing, VEGF may find use as a neural survival factor for degenerative retinal pathologies.

## Materials and Methods

### Animals

Adult *VEGF-lacZ* mice (from Andreas Nagy, University of Toronto, Canada) [28] and adult *VEGFR2-lacZ* mice [31] (generous gift of Victoria Bautch, UNC-Chapel Hill, NC) were used for localization of VEGF and VEGFR2, respectively. Adult C57BL/6J mice were used for analysis of VEGFR expression and subsequent VEGF-induced photoreceptor survival (JAX Laboratories). For *in vivo* neutralization of VEGF, adult CD-1 mice 6-8 weeks old (Charles River Laboratories) were injected with either 1×10^∧10^ viral particles (V.P.) Ad-CMV-null (control) or 2.5×10^∧9^ V.P. Ad-CMV-sFlt1 (murine form consisting of the first three IgG repeats) [25], [77](Q·Biogene) on day 0. sFlt1 expression was confirmed by ELISA (R&D Systems). Ad-CMV-null and Ad-CMV-sFlt1 did not contain an Ig Fc region. Absence of systemic immune response was confirmed by the absence of leukocyte and platelet activation (data not shown). Circulating levels of sFlt1 of ∼200 ng/ml were measured on the plasma fraction seven days post-infection and were sustained for at least 21 days. Ad-null infected mice showed no detectable sFlt1. All animal protocols were approved by the Schepens Eye Research IACUC.

### Fluorescein-dextran perfusion

Anesthetized mice were perfused through the aorta with fluorescein dextran 2×10^∧6^ m.w. (Sigma) 50 mg/ml in 4% paraformaldehyde in PBS. Eyes were then removed, fixed in 4% paraformaldehyde at 4°C overnight and processed for flat-mount and cryosections.

### Immunohistochemistry

For *VEGF-lacZ* and *VEGFR2-lacZ* staining, sections and flat-mounted retinas were stained for LacZ using the in situ β-galactosidase kit, according to the manufacturer's instructions (Stratagene). Flat-mounted retina, cryosections and photoreceptor explants were incubated overnight at 4°C with rabbit anti-mouse VEGFR2 T1014 (1∶500, a gift from Dr. Brekken, University of Texas Southwestern Medical Center, Dallas, TX) [78], [79], mAb anti-CRALBP (1∶500, Abcam), rabbit anti-VEGFR1 (1∶100; Santa Cruz), rabbit anti-GFAP (1∶300, Sigma), mAb anti-GFAP (1∶250, Millipore), rabbit anti-collagen type IV (1∶400, Abcam), rabbit anti-NG2 (1∶300; Millipore), mAb anti-GS (1∶250, Chemicon), rabbit anti-recoverin (1∶500, Chemicon). Secondary antibodies included biotinylated anti-rabbit or anti-mouse antibodies (Vector Laboratories) and Cy5-, Cy3- or FITC-conjugated antibody (Jackson ImmunoResearch Laboratories). Some antibodies were visualized using 3, 3′-diaminobenzidine (ABC kit; Vector Laboratories). ECs were detected using FITC-conjugated *Griffonia simplicifolia* isolectin B4 (Vector Laboratories). Cell nuclei were identified by DAPI labeling. Incubation using rabbit or mouse IgG as a primary antibody was conducted as a negative control.

### Quantification of retinal vessel perfusion

Retinal vascular perfusion was measured by comparing the number of type IV collagen-positive vessels of the inner retina (GCL) to the number of FITC positive vessels on 3 serial cryosections separated by 150 µm.

### Histology and electron microscopy

Tissues were prepared for TEM as previously described [20]. Ultrathin sections were treated with uranyl acetate and visualized using a Phillips 410 transmission electron microscope.

### Quantification of INL and ONL thickness

Semi-thin sections were cut along the vertical meridian passing through the optic nerve and stained with Richardson's stain for light microscopy. Three serial sections (100 µm apart) were quantified per animal. On each section, photographs were taken at 600 µm and 1800 µm from the optic nerve on each side. Three measurements were made per photographs. For each animal, the ONL and INL thickness was obtained by averaging all 36 measurements.

### Quantification of choriocapillaris fenestrations

Endothelial fenestrations were quantified from electron micrographs of the peripheral region of the RPE-choroid (approximately 800 µm from the optic nerve) at a magnification of 10,400×. Only the RPE-side vessel wall was taken into consideration. Fenestrations were counted on 13–15 fields showing continuous choriocapillaris lumen with a length of 10 µm each.

### VEGFR2 activation status

Adult mouse retinas were dissected, pooled, lysed, and protein concentration was determined using a BCA assay (BioRad). VEGFR2 immunoprecipitation and phospho-tyrosine blot was performed as previously described [3]. As a control, porcine aortic cells overexpressing human VEGFR2 (PAE-VEGFR2) were serum-starved overnight and incubated with 50 ng/mlVEGF165 (R&D Systems) for five min. Lysates were then collected and used for immunoprecipitation.

### Fluorescein angiography

Fluorescein angiography was performed after intraperitoneal injection of 60 mg/kg of 25% fluorescein sodium (Akorn). Photographs were taken using the Topcon/Imagenet system (Topcon Medical System) with a preset 20D lens apposed to the fundus camera at regular time intervals (from 1 min to 4 min post I.P injection). Fluorescein leakage was noted as diffuse opacity in the vitreous over-time.

### TUNEL assay

Apoptotic cells were detected using the Dead End HRP kit (Promega) or the Roche In Situ Cell Death Detection TMR red kit (Roche Diagnostic) following the manufacturer's instructions.

### Electroretinography

Mouse ERG was assessed 28 days post adenovirus infection on dark-adapted mice using a UTAS-E3000 recording system (LKC, Technologies, Inc.). After pupil dilatation with 1% tropicamide, 1.5% cyclopentolate, each mouse was placed in front of a Ganzfeld bowl (UTAS3000; LKC Technologies). ERG responses to a series of increasing-intensity light flashes: ±0, +10 and +20-dB were averaged over 10 separate flashes per light intensity.

### Cell culture

The human Müller cell line, MIO-M1 (a gift from G.A. Limb, Institute of Ophthalmology and Moorfields Eye Hospital, London, UK), was maintained in culture at 37°C, 5% CO_2_ in Dulbecco's modified Eagle's medium (DMEM) (Invitrogen Gibco) supplemented with 2 mmol/L glutamine, 100 IU/ml penicillin, 100 µg/ml streptomycin (all from Gibco) and 10% fetal bovine serum (Sigma). Cells were used from passage P16 to P25. MIO-M1 apoptosis was quantified using the Vybrant Apoptosis Assay #2 (Molecular Probes) followed by flow cytometry using a FACSCAN flow cytometer.

### siRNA mediated gene silencing of VEGF

Inhibition of VEGF expression was achieved using an ON-TARGETplus SMARTpool containing four pooled siRNA duplexes (Dharmacon). An unrelated control siRNA pool that lacks identity with known gene targets was used as a control for non-sequence-specific effects. MIO-M1 cells were transfected with siRNA using Dharmafect 1. VEGF in the cell culture supernatant was determined using a Quantikine human VEGF Immunoassay kit (R&D Systems).

### Reverse Transcription-PCR Analysis

Total RNA was isolated from tissue and cells using RNA-Bee solution (IsoText Diagnostic Inc.). Müller cells were isolated from adult rat retinas by density gradient centrifugation as described previously [80] and total RNA isolated using the RNEasy kit (Quiagen). RNA was reverse-transcribed using Superscript III (Invitrogen). Standard PCR was performed with 1 U Taq DNA polymerase (Roche Diagnostics) and 0.2 µM of appropriate primer pair (Table 1). qPCR reactions were performed using the SYBR Green Master mix and the ABI Prism 9700 Sequence Detection System (Applied Biosystems) according to the manufacturer's instructions. For primer sequence, see Table 2.

**Table 1:** RT-PCR primers.

Gene	primer forward (5′-3′)	primer reverse (5′-3′)	Product size
Mouse VEGFR1	gagagcatctataaggcagcggatt	cacgtttacaatgagagtggcagtg	456 bp
Mouse VEGFR2	tacacaattcagagcgatgtgtggt	ctggttcctccaatgggatatcttc	499 bp
Mouse Neuropilin-1	tcaggaccatacaggagatgg	tgacatcccattgtgccaac	619 bp
Mouse Neuropilin-2	agactaccaccccatatcccatgg	ctgccctggtcctcacggatg	421 bp
Mouse GAPDH	gtggcaaagtggagatggttgcc	gatgatgacccgtttggctcc	291 bp
Rat VEGFR1	caagggactctacacttgtc	ccgaatagcgagcagatttc	240 bp
Rat VEGFR2	gccaatgaaggggaactgaaga	ctctgactgctggtgatgctgtc	537 bp
Rat VEGF	gctctcttgggtgcactggac	acggcaatagctgcgctggta	145 bp
Rat Neuropilin-1	ccacagagaagccaaccatt	tgaccctcagtgtacccaca	333 bp
Rat GAPDH	ggtcatccctgagctgaacg	tccgttgtcataccaggaaat	294 bp
Human VEGFR1	caagtggccagaggcatggagtt	gatgtagtctttaccatcctgttg	498 bp
Human VEGF2	gagggcctctcatggtgattgt	tgccagcagtccagcatggtctg	709 bp
Human GAPDH	caaattccatggcaccgtca	ggagtgggtgtcgctgttga	715 bp

**Table 2:** qPCR primers.

Gene	primer forward (5′-3′)	primer reverse (5′-3′)
VEGF	gggcagaatcatcacgaagtg	attggatggcagtagctgcg
Bax	aagctgagcgagtgtctccggcg	gccacaaagatggtcactgtctgcc
Bcl-2	ccgggagaacagggtatgataa	cccactcgtagcccctctg
Bcl-xl	tggagtaaactgggggtcgcatcg	agccaccgtcatgcccgtcagg
GAPDH	cccatcaccatcttccagga	catcgcccacttgattttg

### Isolation of photoreceptor sheets

Sheets of photoreceptors were isolated from adult C57BL/6J mice as described previously [81], [82]. Retinas from 6–8 week old C57BL/6J mice were dissected and flattened by making 4 radial cuts, then placed on a 20% gelatin block secured to a vibratome chuck. Starting at the vitreal surface, sequential sections were cut until the photoreceptor layer was reached then a 200 µm thick section, containing the ONL and IS, was collected. In some experiments the 60 µm thick sections were cultured as ONL explants in the presence or absence of 10 ng/ml VEGF165 (R&D Systems) and apoptotic cells per section were quantified by TUNEL assay.

### Trypan blue exclusion assay

Photoreceptor cells collected by enzymatic digestion (papain) of vibratome-isolated photoreceptor sheets were cultured in 96 well laminin-coated plates (Sigma) at 37°C under 5% CO_2_ in neurobasal medium (Gibco) supplemented with 2% B27 (Gibco), 1% pen/strep (Sigma), 1% glutamine (Sigma) and 0.2% nystatin (Sigma), in the presence or absence of 10 ng/ml VEGF165 (R&D Systems, obtained from the NIH-NCI Preclinical Repository). Twenty-four, 48 or 72 hrs post-plating, 10 µl of trypan blue dye was added for 5 min and the numbers of stained (dead) and unstained (live) cells were counted.

### Statistical analysis

Values are expressed as mean±SD (unless specified); statistical analysis was performed using an unpaired Student t test (***: P<0.001, **: P<0.01, *: P<0.05, ns: P>0.05).

### Online supplemental material

Fig. S1 shows VEGF-expressing astrocytes, characterized by GFAP and β-gal staining, in adult VEGF-LacZ retina flat-mount. Fig. S2 demonstrates normal pericyte coverage of retinal microvessels 14 days after Ad-sFlt1 infection. Fig. S3 demonstrates the absence of GFAP up-regulation in Müller cells after Ad-sFlt1 infection. Fig. S4 shows the successive steps of photoreceptor sheets isolation by vibratome sectioning. Fig. S5 demonstrates the purity of the photoreceptor explants which stain positive for recoverin and lack the Müller cell specific markers, GFAP and glutamine synthetase (GS).

## Supporting Information

Figure S1 Expression of VEGF by astrocytes in adult retina. VEGF expressing cells were detected on retina flat-mounts of adult VEGF-lacZ mice by β-gal staining. Co-staining with GFAP revealed a high number of astrocytes, with processes wrapping the retina vessels, expressing VEGF as shown by the blue staining of their cell bodies (black arrowheads). Some lacZ-positive GFAP-negative cells, presumably pericytes, are observed apposed to the vessel wall (white arrows). Scale bar are 100 µm.
(5.90 MB TIF)

Figure S2 Normal pericyte coverage in microvessels of sFlt1 treated mice. Mice expressing Ad-null or Ad-sFlt1 for 14 days were perfused with h.m.w fluorescein dextran (green), the retinas were dissected, flat mounted and stained for the pericyte marker, NG2 (red). No changes in the association of pericytes with the retinal microvessels were observed (arrows). Scale bar is 50 µm.
(0.93 MB TIF)

Figure S3 Absence of glial activation in the retina of sFlt1-expressing mice. Sections of eyes from mice infected by Ad-null and Ad-sFlt1 for 14 days were stained for the intermediate filament protein, glial fibrillary acidic protein (GFAP). Upregulation of GFAP in the Müller cells endfeet and processes is associated with an ongoing injury response. However, GFAP appeared normally restricted to the astrocytes in the GCL of both Ad-null and Ad-sFlt1 mice. GCL: ganglion cell layer; IPL: inner plexiform layer; INL: inner nuclear layer. Scale is bar 100 µm.
(1.06 MB TIF)

Figure S4 Isolation of photoreceptors sheets. Adult mouse retinas were flat-mounted onto a 20% gelatin block and sectioned along the horizontal plane until the ONL was reached. (A) Photograph of a gelatin block stained by hematoxylin and eosin (H&E) showing that only the photoreceptors (ONL, IS and OS) remain after the inner layers are sectionned. (B) H&E staining of an isolated photoreceptor sheet that will be subsequently used for ONL explant or photoreceptor cell culture. OS: outer segment; IS, inner segment; ONL, outer nuclear layer. Scale bar is 100 µm in A and 50 µm in B.
(0.93 MB TIF)

Figure S5 Verification of the photoreceptor explant purity. ONL explants were co-stained for the photoreceptor marker, recoverin, and the Müller cell markers, GFAP and GS. All cells in the ONL explants were positive for recoverin, confirming the presence of photoreceptors. No GFAP- or GS-positive cells were detected. Some remnants of Müller cell basal processes could be observed (arrows), demonstrating the purity of the photoreceptor sheets. Positive control for GFAP and GS staining consisting of full retina sections were included (data not shown). Scale bar is 20 µm.
(5.04 MB TIF)

## References

[1] Saint-Geniez M, D'Amore PA (2004). Development and pathology of the hyaloid, choroidal and retinal vasculature.. Int J Dev Biol.

[2] Darland DC, Massingham LJ, Smith SR, Piek E, Saint-Geniez M (2003). Pericyte production of cell-associated VEGF is differentiation-dependent and is associated with endothelial survival.. Dev Biol.

[3] Saint-Geniez M, Maldonado AE, D'Amore PA (2006). VEGF expression and receptor activation in the choroid during development and in the adult.. Invest Ophthalmol Vis Sci.

[4] Marneros AG, Fan J, Yokoyama Y, Gerber HP, Ferrara N (2005). Vascular endothelial growth factor expression in the retinal pigment epithelium is essential for choriocapillaris development and visual function.. Am J Pathol.

[5] Stone J, Itin A, Alon T, Pe'er J, Gnessin H (1995). Development of retinal vasculature is mediated by hypoxia-induced vascular endothelial growth factor (VEGF) expression by neuroglia.. J Neurosci.

[6] Alon T, Hemo I, Itin A, Pe'er J, Stone J (1995). Vascular endothelial growth factor acts as a survival factor for newly formed retinal vessels and has implications for retinopathy of prematurity.. Nature Medicine.

[7] Stalmans I, Ng YS, Rohan R, Fruttiger M, Bouche A (2002). Arteriolar and venular patterning in retinas of mice selectively expressing VEGF isoforms.. J Clin Invest.

[8] Ferrara N, Mass RD, Campa C, Kim R (2007). Targeting VEGF-A to treat cancer and age-related macular degeneration.. Annu Rev Med.

[9] Duh EJ, Yang HS, Haller JA, De Juan E, Humayun MS (2004). Vitreous levels of pigment epithelium-derived factor and vascular endothelial growth factor: implications for ocular angiogenesis.. Am J Ophthalmol.

[10] Ambati J, Ambati B, Yoo S, Ianchulev S, Adamis A (2003). Age-related macular degeneration: etiology, pathogenesis, and therapeutic strategies.. Surv Ophthalmol.

[11] Tilton RG, Kawamura T, Chang KC, Ido Y, Bjercke RJ (1997). Vascular dysfunction induced by elevated glucose levels in rats is mediated by vascular endothelial growth factor.. J Clin Invest.

[12] Gragoudas ES, Adamis AP, Cunningham ET, Feinsod M, Guyer DR (2004). Pegaptanib for neovascular age-related macular degeneration.. N Engl J Med.

[13] Brown DM, Kaiser PK, Michels M, Soubrane G, Heier JS (2006). Ranibizumab versus verteporfin for neovascular age-related macular degeneration.. N Engl J Med.

[14] Kim I, Ryan AM, Rohan R, Amano S, Agular S (1999). Constitutive expression of VEGF, VEGFR-1, and VEGFR-2 in normal eyes. [erratum appears in Invest Ophthalmol Vis Sci 2000 Feb;41(2):368].. Investigative Ophthalmology & Visual Science.

[15] Robinson GS, Ju M, Shih SC, Xu X, McMahon G (2001). Nonvascular role for VEGF: VEGFR-1, 2 activity is critical for neural retinal development.. FASEB Journal.

[16] Famiglietti EV, Stopa EG, McGookin ED, Song P, LeBlanc V (2003). Immunocytochemical localization of vascular endothelial growth factor in neurons and glial cells of human retina.. Brain Res.

[17] Vinores SA, Derevjanik NL, Shi A, Vinores MA, Klein DA (2001). Vascular endothelial growth factor (VEGF), transforming growth factor-beta (TGFbeta), and interleukin-6 (IL-6) in experimental herpesvirus retinopathy: association with inflammation and viral infection.. Histol Histopathol.

[18] D'Amore PA (2007). Vascular Endothelial Cell Growth Factor-A. Not Just for Endothelial Cells Anymore.. Am J Pathol.

[19] Schanzer A, Wachs FP, Wilhelm D, Acker T, Cooper-Kuhn C (2004). Direct stimulation of adult neural stem cells in vitro and neurogenesis in vivo by vascular endothelial growth factor.. Brain Pathol.

[20] Maharaj AS, Walshe TE, Saint-Geniez M, Venkatesha S, Maldonado AE (2008). VEGF and TGF-beta are required for the maintenance of the choroid plexus and ependyma.. J Exp Med.

[21] Azzouz M, Ralph GS, Storkebaum E, Walmsley LE, Mitrophanous KA (2004). VEGF delivery with retrogradely transported lentivector prolongs survival in a mouse ALS model.. Nature.

[22] Wang Y, Mao XO, Xie L, Banwait S, Marti HH (2007). Vascular endothelial growth factor overexpression delays neurodegeneration and prolongs survival in amyotrophic lateral sclerosis mice.. J Neurosci.

[23] Spandau UH, Jonas JB (2006). Retinal pigment epithelium tear after intravitreal bevacizumab for exudative age-related macular degeneration.. Am J Ophthalmol.

[24] Weinberger AW, Thiel M, Mohammadi B, Theofylaktopoulos I, Thumann G (2007). Retinal pigment epithelium tears after intravitreal bevacizumab in pigment epithelium detachment.. Am J Ophthalmol.

[25] Maynard SE, Min JY, Merchan J, Lim KH, Li J (2003). Excess placental soluble fms-like tyrosine kinase 1 (sFlt1) may contribute to endothelial dysfunction, hypertension, and proteinuria in preeclampsia.. J Clin Invest.

[26] Sathish S, Arnold JJ (2000). Bilateral choroidal ischaemia and serous retinal detachment in pre-eclampsia.. Clin Experiment Ophthalmol.

[27] Saito Y, Tano Y (1998). Retinal pigment epithelial lesions associated with choroidal ischemia in preeclampsia.. Retina.

[28] Miquerol L, Gertsenstein M, Harpal K, Rossant J, Nagy A (1999). Multiple developmental roles of VEGF suggested by a LacZ-tagged allele.. Dev Biol.

[29] Ozerdem U, Grako KA, Dahlin-Huppe K, Monosov E, Stallcup WB (2001). NG2 proteoglycan is expressed exclusively by mural cells during vascular morphogenesis.. Dev Dyn.

[30] Bignami A, Eng LF, Dahl D, Uyeda CT (1972). Localization of the glial fibrillary acidic protein in astrocytes by immunofluorescence.. Brain Res.

[31] Shalaby F, Rossant J, Yamaguchi TP, Gertsenstein M, Wu X-F (1995). Failure of blood-island formation and vasculogenesis in Flk-1-deficient mice.. Nature.

[32] Bunt-Milam AH, Saari JC (1983). Immunocytochemical localization of two retinoid-binding proteins in vertebrate retina.. J Cell Biol.

[33] Kendall RL, Wang G, Thomas KA (1996). Identification of a natural soluble form of the vascular endothelial growth factor receptor, FLT-1, and its heterodimerization with KDR.. Biochem Biophys Res Commun.

[34] Kamba T, Tam BY, Hashizume H, Haskell A, Sennino B (2006). VEGF-dependent plasticity of fenestrated capillaries in the normal adult microvasculature.. Am J Physiol Heart Circ Physiol.

[35] Venkatesha S, Toporsian M, Lam C, Hanai J, Mammoto T (2006). Soluble endoglin contributes to the pathogenesis of preeclampsia.. Nat Med.

[36] Erickson KK, Sundstrom JM, Antonetti DA (2007). Vascular permeability in ocular disease and the role of tight junctions.. Angiogenesis.

[37] Lewis GP, Fisher SK (2003). Up-regulation of glial fibrillary acidic protein in response to retinal injury: its potential role in glial remodeling and a comparison to vimentin expression.. Int Rev Cytol.

[38] Jeon CJ, Strettoi E, Masland RH (1998). The major cell populations of the mouse retina.. J Neurosci.

[39] Pinto LH, Invergo B, Shimomura K, Takahashi JS, Troy JB (2007). Interpretation of the mouse electroretinogram.. Doc Ophthalmol.

[40] Limb GA, Salt TE, Munro PM, Moss SE, Khaw PT (2002). In vitro characterization of a spontaneously immortalized human Muller cell line (MIO-M1).. Invest Ophthalmol Vis Sci.

[41] Gross A, McDonnell JM, Korsmeyer SJ (1999). BCL-2 family members and the mitochondria in apoptosis.. Genes Dev.

[42] Gilbert RE, Vranes D, Berka JL, Kelly DJ, Cox A (1998). Vascular endothelial growth factor and its receptors in control and diabetic rat eyes.. Lab Invest.

[43] Nishijima K, Ng Y-S, Zhong L, Bradley J, Shubert W (2007). VEGF-A is a survival factor for retinal neurons and a critical neuroprotectant during the adaptive response to ischemic injury.. Am J Path.

[44] Gerhardinger C, Brown LF, Roy S, Mizutani M, Zucker CL (1998). Expression of vascular endothelial growth factor in the human retina and in nonproliferative diabetic retinopathy.. Am J Pathol.

[45] Stitt AW, Simpson DA, Boocock C, Gardiner TA, Murphy GM (1998). Expression of vascular endothelial growth factor (VEGF) and its receptors is regulated in eyes with intra-ocular tumours.. J Pathol.

[46] Fragoso R, Elias AP, Dias S (2007). Autocrine VEGF loops, signaling pathways, and acute leukemia regulation.. Leuk Lymphoma.

[47] Mercurio AM, Bachelder RE, Bates RC, Chung J (2004). Autocrine signaling in carcinoma: VEGF and the alpha6beta4 integrin.. Semin Cancer Biol.

[48] Guan F, Villegas G, Teichman J, Mundel P, Tufro A (2006). Autocrine VEGF-A system in podocytes regulates podocin and its interaction with CD2AP.. Am J Physiol Renal Physiol.

[49] Bryan BA, Walshe TE, Mitchell DC, Havumaki JS, Saint-Geniez M (2007). Coordinated Vascular Endothelial Growth Factor Expression and Signaling During Skeletal Myogenic Differentiation.. Mol Biol Cell.

[50] Maharaj AS, Saint-Geniez M, Maldonado AE, D'Amore PA (2006). Vascular endothelial growth factor localization in the adult.. Am J Pathol.

[51] Lee S, Chen TT, Barber CL, Jordan MC, Murdock J (2007). Autocrine VEGF signaling is required for vascular homeostasis.. Cell.

[52] Culmsee C, Mattson MP (2005). p53 in neuronal apoptosis.. Biochem Biophys Res Commun.

[53] Newman E, Reichenbach A (1996). The Muller cell: a functional element of the retina.. Trends Neurosci.

[54] Seki M, Nawa H, Fukuchi T, Abe H, Takei N (2003). BDNF is upregulated by postnatal development and visual experience: quantitative and immunohistochemical analyses of BDNF in the rat retina.. Invest Ophthalmol Vis Sci.

[55] Carmignoto G, Comelli MC, Candeo P, Cavicchioli L, Yan Q (1991). Expression of NGF receptor and NGF receptor mRNA in the developing and adult rat retina.. Exp Neurol.

[56] Vecino E, Caminos E, Becker E, Martin-Zanca D, Osborne NN, Castellanos BB, Gonzalez M, Nieto Sampedro R (1998). Expression of neurotrophins and their receptors within the glial cells of retina and optic nerve.. Understanding Glia.

[57] Garcia M, Forster V, Hicks D, Vecino E (2002). Effects of muller glia on cell survival and neuritogenesis in adult porcine retina in vitro.. Invest Ophthalmol Vis Sci.

[58] Garcia M, Vecino E (2003). Role of Muller glia in neuroprotection and regeneration in the retina.. Histol Histopathol.

[59] Isenmann S, Kretz A, Cellerino A (2003). Molecular determinants of retinal ganglion cell development, survival, and regeneration.. Prog Retin Eye Res.

[60] Linsenmeier RA (1990). Electrophysiological consequences of retinal hypoxia.. Graefes Arch Clin Exp Ophthalmol.

[61] Shweiki D, Itin A, Soffer D, Keshet E (1992). Vascular endothelial growth factor induced by hypoxia may mediate hypoxia-initiated angiogenesis.. Nature.

[62] Stein I, Neeman M, Shweiki D, Itin A, Keshet E (1995). Stabilization of vascular endothelial growth factor mRNA by hypoxia and hypoglycemia and coregulation with other ischemia-induced genes.. Mol Cell Biol.

[63] Zachary I (2001). Signaling mechanisms mediating vascular protective actions of vascular endothelial growth factor.. American Journal of Physiology - Cell Physiology.

[64] Sondell M, Sundler F, Kanje M (2000). Vascular endothelial growth factor is a neurotrophic factor which stimulates axonal outgrowth through the flk-1 receptor.. European Journal of Neuroscience.

[65] Ng Y-S, Rohan R, Sunday M, deMello DE, D'Amore PA (2001). Differential expression of VEGF isoforms in mouse during development and in the adult.. Dev Dyn.

[66] Roberts WG, Palade GE (1995). Increased microvascular permeability and endothelial fenestration induced by vascular endothelial growth factor.. J Cell Sci.

[67] Yokomori H, Oda M, Yoshimura K, Nagai T, Ogi M (2003). Vascular endothelial growth factor increases fenestral permeability in hepatic sinusoidal endothelial cells.. Liver Int.

[68] Peters S, Heiduschka P, Julien S, Ziemssen F, Fietz H (2007). Ultrastructural findings in the primate eye after intravitreal injection of bevacizumab.. Am J Ophthalmol.

[69] Engerman RL, Pfaffenbach D, Davis MD (1967). Cell turnover of capillaries.. Lab Invest.

[70] Antonelli-Orlidge A, Saunders KB, Smith SR, D'Amore PA (1989). An activated form of transforming growth factor ß is produced by cocultures of endothelial cells and pericytes.. Proc Natl Acad Sci USA.

[71] Hirschi K, Rohovsky SA, D'Amore PA (1998). PDGF, TGF-ß and heterotypic cell-cell interactions mediate the recruitment and differentiation of 10T1/2 cells to a smooth muscle cell fate.. J Cell Biol.

[72] Cogan DG, Kuwabara T (1967). The mural cell in perspective.. Arch Ophthal.

[73] Cogan DG, Toussaint D, Kuwabara T (1961). Retinal vascular patterns. IV. Diabetic retinopathy.. Arch Ophthalmol.

[74] Ramsauer M, D'Amore PA (2007). Contextual role for angiopoietins and TGFbeta1 in blood vessel stabilization.. J Cell Sci.

[75] Darland DC, D'Amore PA (2001). TFGß is required for the formation of capillary-like structures in three-dimensional cocultures of 10T1/2 and endothelial cells.. Angiogenesis.

[76] Uemura A, Ogawa M, Hirashima M, Fujiwara T, Koyama S (2002). Recombinant angiopoietin-1 restores higher-order architecture of growing blood vessels in mice in the absence of mural cells.. J Clin Invest.

[77] Kuo CJ, Farnebo F, Yu EY, Christofferson R, Swearingen RA (2001). Comparative evaluation of the antitumor activity of antiangiogenic proteins delivered by gene transfer.. Proc Natl Acad Sci U S A.

[78] Brekken RA, Overholser JP, Stastny VA, Waltenberger J, Minna JD (2000). Selective inhibition of vascular endothelial growth factor (VEGF) receptor 2 (KDR/Flk-1) activity by a monoclonal anti-VEGF antibody blocks tumor growth in mice.. Cancer Res.

[79] Feng D, Nagy JA, Brekken RA, Pettersson A, Manseau EJ (2000). Ultrastructural localization of the vascular permeability factor/vascular endothelial growth factor (VPF/VEGF) receptor-2 (FLK-1, KDR) in normal mouse kidney and in the hyperpermeable vessels induced by VPF/VEGF-expressing tumors and adenoviral vectors.. J Histochem Cytochem.

[80] Gerhardinger C, Costa MB, Coulombe MC, Toth I, Hoehn T (2005). Expression of acute-phase response proteins in retinal Muller cells in diabetes.. Invest Ophthalmol Vis Sci.

[81] Fontaine V, Kinkl N, Sahel J, Dreyfus H, Hicks D (1998). Survival of purified rat photoreceptors in vitro is stimulated directly by fibroblast growth factor-2.. J Neurosci.

[82] Mohand-Said S, Hicks D, Simonutti M, Tran-Minh D, Deudon-Combe A (1997). Photoreceptor transplants increase host cone survival in the retinal degeneration (rd) mouse.. Ophthalmic Res.

