# Supplementary figures and images for: Endogenous VEGF Is Required for Visual Function: Evidence for a Survival Role on Müller Cells and Photoreceptors
Source: PLoS One. 2008 Nov 3;3(11):e3554. doi: 10.1371/journal.pone.0003554 (PMC2571983; doi:10.1371/journal.pone.0003554)

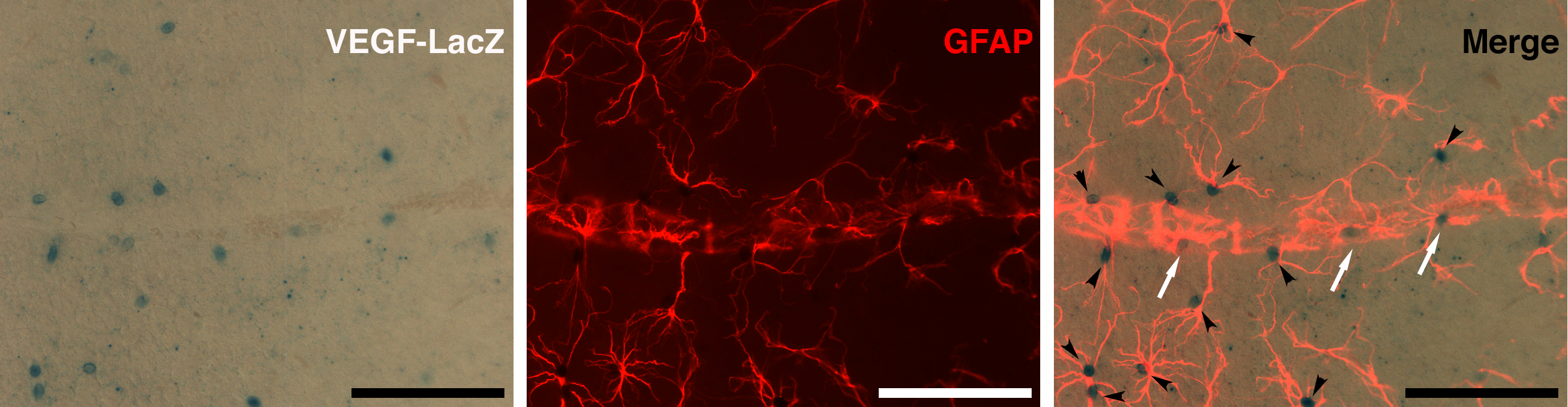

Supplement: Figure S1 — Expression of VEGF by astrocytes in adult retina. VEGF expressing cells were detected on retina flat-mounts of adult VEGF-lacZ mice by β-gal staining. Co-staining with GFAP revealed a high number of astrocytes, with processes wrapping the retina vessels, expressing VEGF as shown by the blue staining of their cell bodies (black arrowheads). Some lacZ-positive GFAP-negative cells, presumably pericytes, are observed apposed to the vessel wall (white arrows). Scale bar are 100 µm. (5.90 MB TIF) [file pone.0003554.s001.tif]

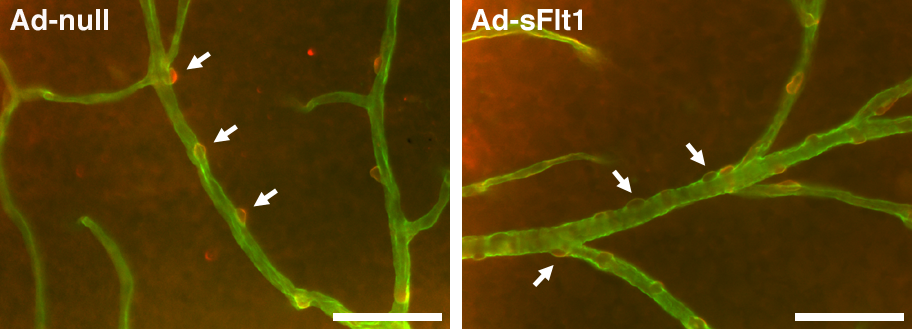

Supplement: Figure S2 — Normal pericyte coverage in microvessels of sFlt1 treated mice. Mice expressing Ad-null or Ad-sFlt1 for 14 days were perfused with h.m.w fluorescein dextran (green), the retinas were dissected, flat mounted and stained for the pericyte marker, NG2 (red). No changes in the association of pericytes with the retinal microvessels were observed (arrows). Scale bar is 50 µm. (0.93 MB TIF) [file pone.0003554.s002.tif]

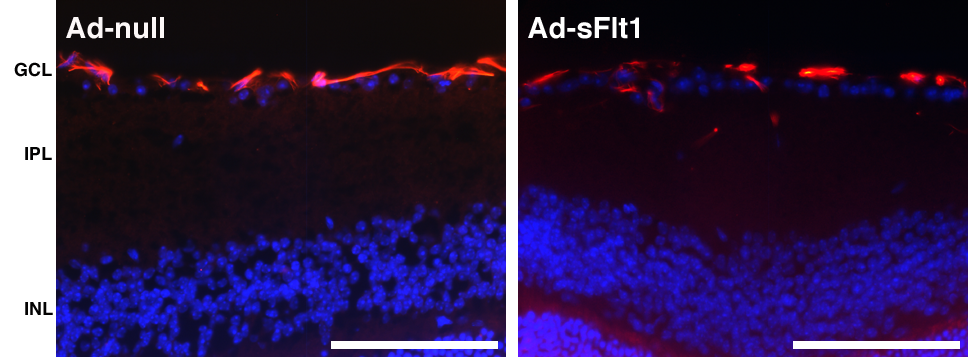

Supplement: Figure S3 — Absence of glial activation in the retina of sFlt1-expressing mice. Sections of eyes from mice infected by Ad-null and Ad-sFlt1 for 14 days were stained for the intermediate filament protein, glial fibrillary acidic protein (GFAP). Upregulation of GFAP in the Müller cells endfeet and processes is associated with an ongoing injury response. However, GFAP appeared normally restricted to the astrocytes in the GCL of both Ad-null and Ad-sFlt1 mice. GCL: ganglion cell layer; IPL: inner plexiform layer; INL: inner nuclear layer. Scale is bar 100 µm. (1.06 MB TIF) [file pone.0003554.s003.tif]

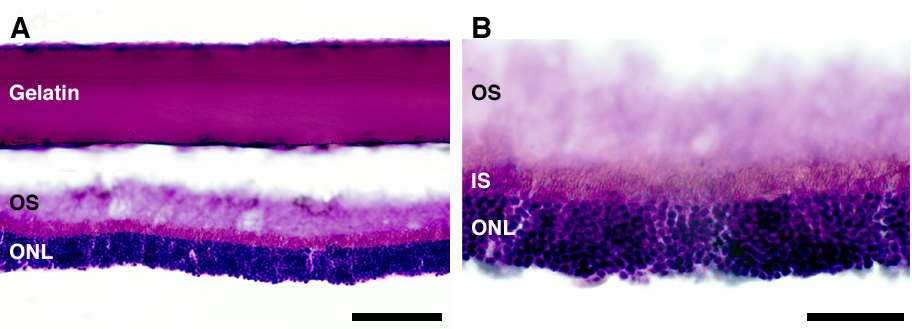

Supplement: Figure S4 — Isolation of photoreceptors sheets. Adult mouse retinas were flat-mounted onto a 20% gelatin block and sectioned along the horizontal plane until the ONL was reached. (A) Photograph of a gelatin block stained by hematoxylin and eosin (H&E) showing that only the photoreceptors (ONL, IS and OS) remain after the inner layers are sectionned. (B) H&E staining of an isolated photoreceptor sheet that will be subsequently used for ONL explant or photoreceptor cell culture. OS: outer segment; IS, inner segment; ONL, outer nuclear layer. Scale bar is 100 µm in A and 50 µm in B. (0.93 MB TIF) [file pone.0003554.s004.tif]

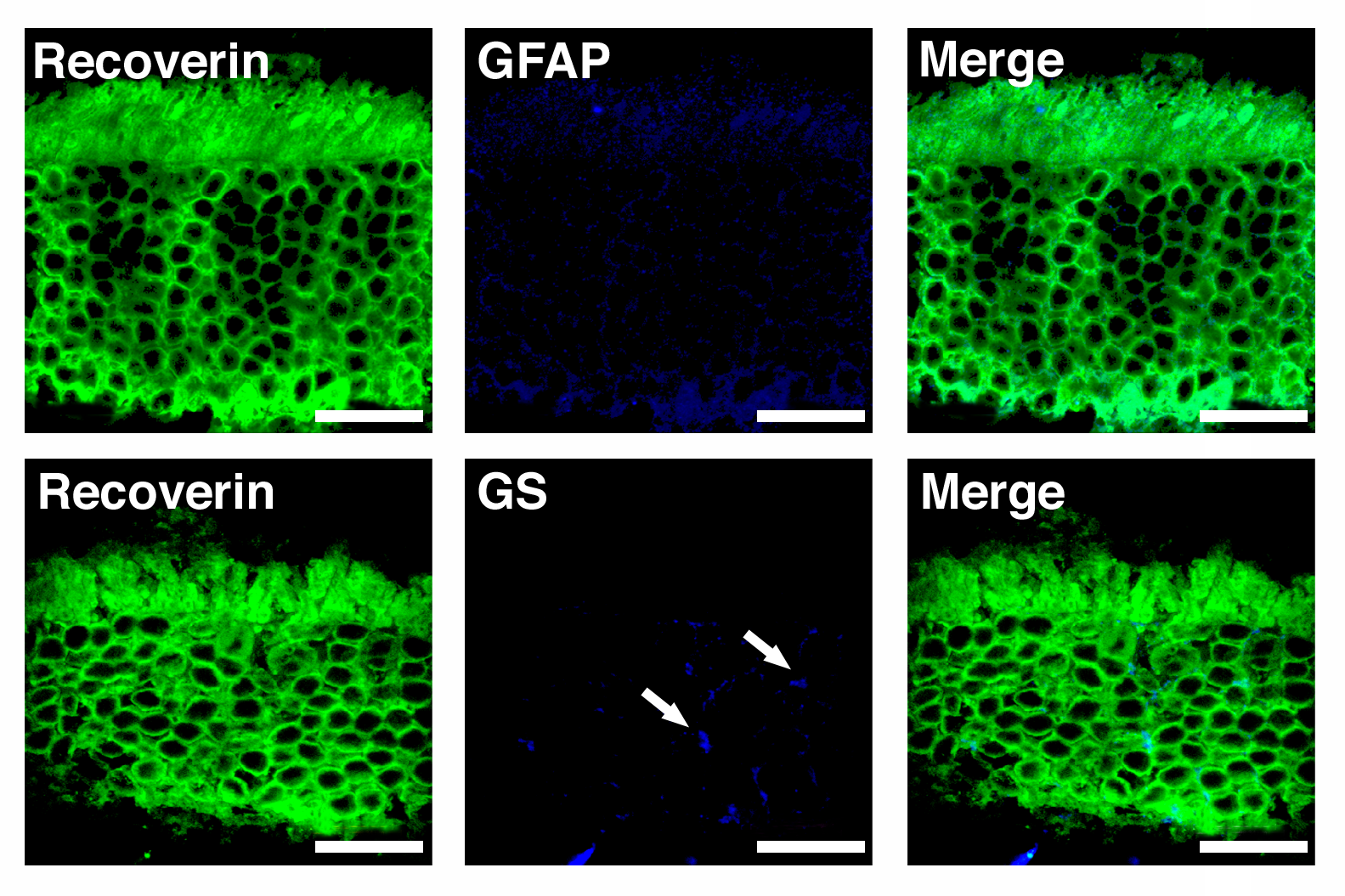

Supplement: Figure S5 — Verification of the photoreceptor explant purity. ONL explants were co-stained for the photoreceptor marker, recoverin, and the Müller cell markers, GFAP and GS. All cells in the ONL explants were positive for recoverin, confirming the presence of photoreceptors. No GFAP- or GS-positive cells were detected. Some remnants of Müller cell basal processes could be observed (arrows), demonstrating the purity of the photoreceptor sheets. Positive control for GFAP and GS staining consisting of full retina sections were included (data not shown). Scale bar is 20 µm. (5.04 MB TIF) [file pone.0003554.s005.tif]
